# Oncolytic virotherapy – *in vivo veritas*

**DOI:** 10.18632/oncotarget.26364

**Published:** 2018-11-20

**Authors:** Iris Kemler, Claudia Neuhauser, David Dingli

**Affiliations:** David Dingli: Department of Molecular Medicine, Mayo Clinic, Rochester, MN, USA; Division of Hematology and Department of Internal Medicine, Mayo Clinic, Rochester, MN, USA

**Keywords:** molecular imaging, quantitation, correlations, population, virus titer

The goal of oncolytic virotherapy is to use the tools of recombinant DNA technology to modify viruses and render them powerful agents that can selectively infect, replicate and kill tumor cells while leaving normal cells unharmed [1]. The natural ability of many viruses to induce cell lysis can be enhanced through the expression of specific therapeutic genes that render tumor cells either sensitive to a pro-drug [2] or radiation (e.g. sodium iodide symporter (NIS) in combination with alpha and beta particle emitting isotopes [3, 4]) or increase the immune response against the tumor [5]. Although tentative examples from mice [6] and humans [7] suggest that oncolytic viruses can eradicate tumors, many barriers to success exist, including (i) access of the virus to the tumor, (ii) inhibition of spread of the virus in the tumor, (iii) loss of contact of specific parts of the tumor from areas of infection, (iv) the immune response to the virus and virus infected cells that can halt the process.

An attractive aspect of oncolytic virotherapy is the possibility that the target itself amplifies the therapy, leading to ongoing infection and spread of the virus in the tumor hopefully leading to its eradication. However, determining whether the oncolytic actually reached tumor sites, established infection and spread throughout the tumor in the *in vivo* setting is difficult. For this reason, an oncolytic measles virus (MV) that expresses the thyroidal sodium iodide symporter (MV-NIS) was generated on the premise that infected tumor cells will be able to express NIS and concentrate radioactive isotopes which allow serial non-invasive imaging of the virus infected cells [3].

**Figure 1:**
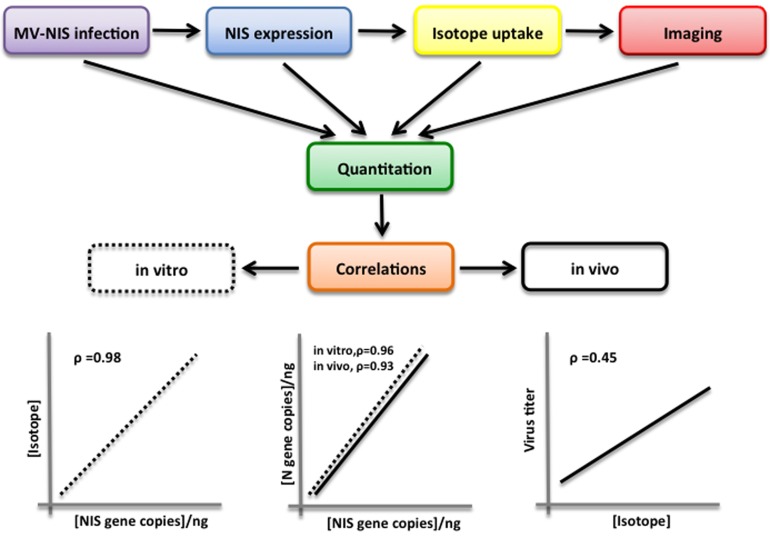
Schematic representation of experimental approach Virus infected cells express NIS and concentrate isotope that can be quantitatively measured in a gamma counter (*in vitro*) or using microSPECT/CT imaging (*in vivo*). Virus mediated N and NIS gene expression as well as virus propagation (virus titer) was quantitated. Correlations were established between virus gene expression, isotope uptake and virus titer *in vitro* and *in vitro*.

Understanding the *in vivo* dynamics of the virus and tumor cell populations is essential to optimize strategies for these emerging technologies. A critical step is the development of quantitative empirical and theoretical approaches to determine population dynamics and inform mathematical models of such dynamics. In order to address this problem, we recently tested the hypothesis that measles virus mediated NIS expression and isotope uptake in tumors can be used to monitor the virus population *in vivo*. Intrinsic to this hypothesis is the assumption that isotope uptake would be proportional to the viable virus present in the tumor at that specific time [8]. In principle, the hypothesis is simple (Figure 1): infected cells express NIS and concentrate isotope that can be quantitatively measured using microSPECT/CT imaging. We postulated that the amount of isotope in the tumor will correlate with the viable virus recovered from the tumor. However, proving such a relationship requires considerable correlative work *in vitro* and *in vivo*. *In vitro* studies of MV-NIS infection of tumor cells showed that the level of NIS expression and isotope uptake correlated well with viral gene expression and viable virus titer. We used two different virus specific genes to understand the dynamics of the virus itself: the ‘N’ gene as well as ‘NIS’. The level of expression of the two genes was highly correlated (ρ=0.96). *In vitro* isotope uptake was also highly correlated with NIS gene expression at each specific time point (ρ=0.979). *In vivo* studies in mice showed similar results with respect to the correlation between viral ‘N’ and ‘NIS’ gene expression implying that *in vivo*, the virus is behaving in a similar way to the *in vitro* scenario. Isotope uptake in the tumor also correlated well with viral gene expression (ρ=0.68). Isotope uptake also had a positive correlation with the viable virus isolated from the tumors (ρ=0.45).

Viral gene copy number changes were observed over the time course of the experiment. Starting with low numbers of copies, these increased with time, implying amplification of the virus. As expected from mathematical models [9], RNA copy numbers experienced oscillatory behavior, which is also observed using reporter systems [10]. Presumably these oscillations are the result of waves of virus propagation followed by cell killing that transiently reduces the target cell population. SPECT/CT imaging was unable to resolve these oscillations likely due to the relatively low level uptake of the isotope. More sensitive approaches such as fluorescence may allow higher resolution, although the approach is less quantitative and does not lend itself to translation in larger animals or humans.

The correlations *in vivo* were inferior to what we observed *in vitro* and this is likely to a number of variables including anisotropies in the distribution of the virus-infected cells that may reduce the sensitivity of the imaging. We showed that in the mice injected with the oncolytic viruses, isotope uptake in the tumor xenografts due to NIS expression was significantly higher than in the controls or background. However, we do not know the minimum number of infected cells that are needed in one focus to enable resolution of the signal via imaging. Is it better to have a few infected cells that express high levels of the reporter gene or is it superior to have more cells express the isotope but at a lower level? We also do not know how many foci of infection are present in the tumor at any time. Answering these questions will be important and likely require the use of multiple modalities of imaging that complement each other without disturbing the cells *in vivo*. We intend to address this problem using a combination of radionuclide as well as fluorescence imaging since the latter can provide single cell resolution if tumor cells are implanted under dorsal skin fold chambers. In this way, we hope that imaging will become truly quantitative and one can determine the population of virus infected tumor cells and its evolution in time from serial radionuclide imaging.
